# Patient safety management activities partially mediate nursing competences and patient safety culture in Vietnam

**DOI:** 10.1371/journal.pone.0345533

**Published:** 2026-07-13

**Authors:** Binh Ho Duy, Phuc Dang Thi Thanh, Nhi Vo Thi, Yen Ho Thi My, Nguyen Nguyen Binh Thao, Quyen Tran Thi Kim, Lan Duong Thi Ngoc

**Affiliations:** 1 University of Medicine and Pharmacy, Hue University, Hue, Vietnam; 2 The University of Danang - School of Medicine and Pharmacy, Da Nang, Vietnam; Universiti Sains Malaysia - Kampus Kesihatan, MALAYSIA

## Abstract

**Background:**

Patient safety culture is a key determinant of healthcare quality, yet evidence remains limited on how nurses’ person-centered care competence and patient safety competence relate to patient safety culture through patient safety management activities, particularly in low- and middle-income settings.

**Objective:**

To describe levels of person-centered care competence, patient safety competence, patient safety management activities, and patient safety culture among nurses in central Vietnam, and to examine their direct and indirect relationships using a mediation model.

**Methods:**

A multicenter cross-sectional study was conducted among 1,036 nurses from five tertiary hospitals in central Vietnam (response rate 99.6%). Person-centered care competence was measured using P-CAT, patient safety competence using H-PEPSS, patient safety management activities using PSMA, and patient safety culture using HSOPSC. Descriptive statistics, Pearson correlations, and multivariable linear regression analyses were performed. Mediation effects were examined using bias-corrected bootstrapping with 5,000 resamples.

**Results:**

Mean scores indicated moderate-to-high levels of person-centered care competence (3.77 ± 0.36), patient safety competence (4.23 ± 0.36), and patient safety management activities (4.44 ± 0.35), while patient safety culture was moderate (3.93 ± 0.35). All variables were positively correlated, with the strongest association observed between person-centered care competence and patient safety culture (r = 0.49, p < 0.001). In adjusted regression analyses, person-centered care competence and patient safety competence were independently associated with patient safety management activities (β = 0.149 and β = 0.274; both p < 0.001). Patient safety management activities were significantly associated with patient safety culture (β = 0.102, p < 0.001). Bootstrapped analyses showed significant partial mediation through patient safety management activities for both person-centered care competence (indirect β = 0.0265, 95% CI 0.0143–0.0404) and patient safety competence (indirect β = 0.0497, 95% CI 0.0304–0.0711), suggesting that frontline safety behaviors may represent a pathway linking competencies to patient safety culture.

**Conclusions:**

Higher person-centered care competence and patient safety competence were associated with stronger patient safety culture, partly through increased engagement in patient safety management activities. These findings highlight the potential role of patient safety management activities as a mechanism linking competencies to patient safety culture. However, causal relationships cannot be inferred due to the cross-sectional design.

## Introduction

Patient safety culture (PSC) is a critical determinant of healthcare quality and patient outcomes. Strong PSC has been consistently associated with reduced adverse events, improved clinical performance, enhanced organizational learning, and greater patient trust [1–5]. Nurses play a central role in patient safety because of their continuous presence at the bedside and their responsibility for coordinating complex clinical care processes across shifts and disciplines.

Person-centered care (PCC) is increasingly recognized as a core component of high-quality and safe healthcare delivery. Contemporary evidence indicates that PCC contributes to safer care by improving communication, patient engagement, and shared decision-making, thereby reducing preventable harm [6], [7], [8]. Competence in delivering PCC among nurses has been associated with better adherence to safety practices and improved patient experiences in recent international studies.

Patient safety competence (PSCp), encompassing knowledge, skills, and attitudes related to teamwork, communication, risk management, and human factors, is essential for preventing adverse events. Recent nursing research has shown that higher PSCp is associated with improved safety behaviors and safety outcomes in hospital settings [9–11]. These findings support the view that competence alone is insufficient unless translated into routine safety practices.

Patient safety management activities (PSMA) represent observable safety behaviors performed by nurses in daily practice, including patient identification, medication safety, infection prevention, and fall prevention. Behavioral and organizational safety theories propose that individual competencies influence safety-related behaviors, which subsequently shape collective safety climate and culture [12,13]. Recent empirical studies in healthcare settings have further supported the role of frontline safety behaviors as proximal determinants of PSC.

Although previous studies have examined the associations between nursing competencies and patient safety culture, the underlying mechanisms through which these competencies are translated into practice remain insufficiently understood. In particular, the mediating role of patient safety management activities as a behavioral pathway has received limited empirical attention. Moreover, most existing evidence has been generated in high-income countries, with limited data from low- and middle-income settings such as Vietnam, where healthcare systems operate under different organizational and resource constraints. Therefore, this study contributes by examining a theory-informed mediation model that clarifies how nursing competencies are operationalized into patient safety management activities and subsequently associated with patient safety culture. Strengthening frontline safety practices has been identified as a global priority in patient safety initiatives [4,5]. Accordingly, this study was guided by a theory-informed conceptual framework that integrates individual competencies, frontline safety behaviors, and organizational PSC. This framework draws on three complementary theoretical perspectives. First, the Theory of Planned Behavior posits that individual knowledge, attitudes, and perceived behavioral control shape the likelihood of behavioral enactment, providing a foundation for linking professional competence to observable safety practices [12]. Second, safety climate and safety performance models conceptualize frontline safety behaviors as proximal determinants through which individual characteristics influence collective safety outcomes [13]. Third, Donabedian’s structure–process–outcome model frames professional competence as a structural attribute that affects care processes and, ultimately, organizational outcomes such as PSC [14].

Within this integrated framework, nurses’ PCC competence and PSCp are conceptualized as distal individual determinants. These competencies are expected to influence the consistent enactment of frontline PSMA, which represent proximal safety behaviors embedded in daily nursing practice. Through repeated and reliable performance of PSMA, shared perceptions, norms, and expectations related to patient safety are reinforced, contributing to the development and maintenance of organizational PSC. This pathway is consistent with systems-based patient safety theory, which emphasizes that individual competence must be translated into reliable processes of care to reduce harm in complex healthcare systems [15]. Thus, PSMA is positioned as a theoretically grounded behavioral mediator rather than a purely statistical construct. In this framework, competencies are conceptualized as structural attributes, patient safety management activities as behavioral processes, and patient safety culture as an organizational outcome. This clarifies the theoretical rationale for modeling mediation as a process linking competence to outcome.

Therefore, this study aimed to (1) describe the levels of PCC competence, PSCp, PSMA, and PSC among nurses in central Vietnam, and (2) examine direct and indirect relationships among these factors using a mediation model in which person-centered care competence and PSCp influence PSC both directly and indirectly through PSMA.

## Materials and methods

### Study design and reporting guideline

A multicenter cross-sectional study was conducted. This manuscript was prepared in accordance with the Strengthening the Reporting of Observational Studies in Epidemiology (STROBE) guidelines for cross-sectional studies [16]. Due to the cross-sectional design, causal relationships cannot be established. The mediation analysis should therefore be interpreted as indicating indirect associations rather than causal pathways.

### Study setting and participants

The study was conducted among nurses working in clinical departments of five tertiary (Grade I) hospitals in central Vietnam. To ensure institutional anonymity and comply with journal requirements, the names of participating hospitals are not disclosed. Eligible participants were registered nurses providing direct patient care and having at least six months of clinical experience at their current workplace. Nurses in administrative positions without routine clinical duties were excluded.

### Sample size determination

A priori sample size estimation was performed using G*Power version 3.1 for a multiple regression model. The calculation assumed a significance level of α = 0.05, statistical power (1 − β) = 0.8, and a small effect size (f² = 0.02), consistent with recommendations for behavioral and health services research. The minimum required sample size was 945. To account for potential non-response, 10% was added, resulting in a target sample size of 1,040 nurses [17]. A total of 1,036 valid questionnaires were included in the final analysis.

### Sampling procedure

A stratified random sampling method with proportional allocation was employed. Hospitals were first selected using convenience sampling due to feasibility and access considerations, which may limit the representativeness of the sample at the institutional level. Within each hospital, the number of nurses invited to participate was determined proportionally based on the total number of eligible nurses using Cochran’s formula for sample allocation in stratified sampling designs [18]. Within each clinical department, nurses were selected using systematic random sampling from staff lists provided by the nursing departments.

### Data collection procedures

Data collection was carried out from June 2024 to December 2024 in collaboration with the nursing departments of participating hospitals. Surveys were administered in paper format during two to three visits to each clinical department to ensure inclusion of nurses across different shifts. Participants were informed about the study objectives, procedures, voluntary nature of participation, and confidentiality measures. Written informed consent was obtained prior to participation. Each questionnaire required approximately 15–20 minutes to complete. Completed questionnaires were reviewed on-site to minimize missing data. Completed questionnaires were reviewed on-site during data collection to ensure completeness. As a result, missing data were minimal. A very small number of questionnaires with incomplete responses were excluded prior to analysis. After this process, missing data were negligible across all study variables (all < 1%). Therefore, complete-case analysis was applied in all statistical models.

### Measurement instruments

Instruments not previously available in Vietnamese (P-CAT, H-PEPSS, and PSMA scale) were translated and culturally adapted following the World Health Organization (WHO) process of translation and adaptation of instruments, including forward translation, expert panel review, back-translation, pretesting, and cognitive interviewing [19,20]. A pilot test was conducted with a sample of nurses to assess clarity and comprehensibility, and minor revisions were made accordingly.

### Person-centered care competence

PCC competence was measured using the Person-Centred Care Assessment Tool (P-CAT), originally developed by Edvardsson et al. The instrument consists of 13 items across three domains: personalized care, organizational support, and environmental accessibility. Items are rated on a 5-point Likert scale (1 = strongly disagree to 5 = strongly agree), with five negatively worded items reverse-coded. Total scores range from 13 to 65, with higher scores indicating greater PCC competence [21]. In the present study, Cronbach’s alpha for the P-CAT was 0.662 (pilot study: 0.772). Although slightly below the conventional 0.70 threshold, this level is considered acceptable in cross-cultural adaptation studies and exploratory research contexts. McDonald’s ω was 0.712, indicating acceptable internal consistency reliability.

### Patient safety competence

PSCp was assessed using the Health Professional Education in Patient Safety Survey (H-PEPSS), developed by Ginsburg et al. The H-PEPSS comprises 23 items across six domains, including teamwork, communication, risk management, human and environmental factors, recognition of adverse events, and safety culture. Responses are rated on a 5-point Likert scale (1 = strongly disagree to 5 = strongly agree), with higher scores indicating greater PSCp [9]. Cronbach’s alpha in this study was 0.898 (pilot study: 0.918) and McDonald’s ω was 0.910.

### Patient safety management activities

PSMA were measured using a 24-item PSMA scale, originally developed and validated among hospital nurses by Park and Kim [22]. The scale covers six domains: patient identification, communication, high-alert medication management, surgical and procedural verification, infection prevention, and fall prevention. Items are rated on a 5-point Likert scale (1 = never to 5 = always), with higher scores indicating more frequent engagement in safety management activities. In the present study, the PSMA demonstrated good internal consistency, with a Cronbach’s alpha of 0.863 (pilot study: 0.910) and McDonald’s ω of 0.893. The conceptual domains of the PSMA are consistent with internationally recognized nursing safety activity frameworks, including the World Health Organization patient safety curriculum and evidence-based hospital safety practices [23,24].

### Patient safety culture

PSC was assessed using the Hospital Survey on PSC (HSOPSC), developed by the Agency for Healthcare Research and Quality (AHRQ). The HSOPSC consists of 42 items across 12 dimensions and is rated on a 5-point Likert scale. Eighteen items are reverse-coded. Positive response rates were calculated following AHRQ guidelines, and mean scores were used for regression analyses. The Vietnamese version of the HSOPSC was translated by Ho Chi Minh city Department of Health and has been officially authorized for public use in Vietnam. This version has demonstrated acceptable reliability and validity in hospital settings. In this study, the overall Cronbach’s alpha for the HSOPSC was 0.889 [25] and McDonald’s ω was 0.911.

All instruments were translated and culturally adapted following established guidelines, including forward translation, expert panel review, back-translation, and pilot testing to ensure clarity and contextual appropriateness. Internal consistency reliability was assessed using both Cronbach’s alpha and McDonald’s omega coefficients. As the primary aim of this study was to examine relationships among constructs rather than to validate the measurement instruments, confirmatory factor analysis (CFA) was not conducted.

### Statistical analysis

Data were analyzed using SPSS software version 20.0 (IBM, Armonk, NY, USA). Descriptive statistics were used to summarize participant characteristics and study variables. Internal consistency reliability was assessed using Cronbach’s alpha coefficients. Pearson correlation analysis was conducted to examine bivariate associations among study variables. Although Likert-scale data are ordinal, they were treated as continuous variables based on approximate normal distribution and common practice in behavioral research. Pearson correlation was therefore considered appropriate for the analysis. This approach is consistent with previous methodological studies supporting the use of parametric analyses for Likert-scale data.

Potential clustering by hospital and clinical unit was assessed using intraclass correlation coefficients (ICCs) derived from random-intercept mixed models. ICC values were close to zero, suggesting negligible clustering effects. Accordingly, standard multivariable linear regression models were used for the primary analyses. To test the hypothesized conceptual framework, PCC competence (P-CAT) and PSCP (H-PEPSS) were entered as predictors of PSMA in Model 1. In Model 2, PCC competence and PSCp were entered as predictors of patient safety culture (HSOPSC). In Model 3, PSMA was added to Model 2 to examine its mediating role. Sociodemographic and professional variables were included as covariates in all regression models, including age, gender, clinical experience, education level, working department, weekly working hours, night shift frequency, job satisfaction, patient safety training, experience with patient safety incidents, and incident reporting. These variables were selected based on prior literature on patient safety culture and nursing performance [1,2], which has identified demographic characteristics, workload-related factors, and safety-related experiences as important determinants of safety behaviors and perceptions of patient safety culture, as well as their relevance in hospital nursing practice. Standardized beta coefficients (β), p-values, R², F statistics and variance inflation factors (VIF) were reported [26].

The distribution of variables was examined prior to analysis. Although normality tests were statistically significant, the data were considered approximately normally distributed given the large sample size. The normality of residuals was assessed using histograms and normal probability (P–P) plots, which indicated acceptable distribution.

Multicollinearity was assessed using tolerance and variance inflation factor (VIF), with all VIF values below 5, indicating no significant multicollinearity.

Mediation analyses were conducted using bootstrapping with 5,000 resamples to estimate indirect effects and 95% bias-corrected confidence intervals. Indirect effects were considered statistically significant if the confidence interval did not include zero [27]. Statistical significance was set at p < 0.05 (two-tailed).

### Ethical considerations

The study was approved by the Ethics Committee of Hue University of Medicine and Pharmacy (No. H2024/022). Permission was obtained from participating hospitals prior to data collection. Participation was voluntary and anonymous. Written informed consent was obtained from all participants before enrollment. All data were collected and analyzed in a manner that ensured confidentiality and anonymity throughout the study.

## Results

The general and work-related characteristics of the participants are presented in **Table 1**. A total of 1,036 nurses were included in the analysis, yielding a response rate of 99.6%. The mean age of participants was 35.41 ± 6.45 years (range: 22–56), and the majority were older than 30 years (77.5%). Most participants were female (89.8%), of Kinh ethnicity (97.3%), and reported no religious affiliation (92.9%). Approximately half of the nurses held a diploma or associate degree (50.9%), while 49.1% had a bachelor’s degree or higher. Most participants were married (82.8%) and worked as staff nurses (92.8%). The mean duration of clinical experience was 12.26 ± 6.31 years, and the mean number of years working in the current department was 10.28 ± 6.00 years. The average weekly working time was 52.67 ± 13.89 hours, with 62.2% working more than 40 hours per week. Nurses worked a mean of 6.26 ± 3.29 night shifts per month. Most participants reported being satisfied with their work (80.1%). Nearly all nurses had experience with patient safety education (99.9%) and PCC education (99.2%). The majority reported experience in patient safety practice (99.5%) and PCC practice (98.0%). A high proportion had experienced patient safety incidents (91.6%), and 66.0% had reported such incidents. Most participants indicated that incident reporting was encouraged in their workplace (98.2%). Overall, 82.7% rated PSC as good, and 74.8% perceived patient safety in their department as good.

**Table 1:** General and work-related characteristics of the participants (N = 1036).

Characteristics		Frequency (n)	Percentage (%)
	Mean ± SD = 35,41 ± 6,45 (22–56)		
**Age**	≤ 30	233	22.50
	> 30	803	77.50
**Gender**	Male	106	10.20
	Female	930	89.80
**Ethnicity**	Kinh	1008	97.30
	Other	28	2.70
**Religion**	No	962	92.90
	Yes	74	7.10
**Education level**	Diploma/Associate degree	527	50.90
	Bachelor’s degree or higher	509	49.10
**Marital status**	Single	151	14.60
	Married	858	82.80
	Other	27	2.60
**Position**	Staff	961	92.80
	Head nurse	75	7.20
**Years of clinical experience (year)**	Mean ± SD = 12.26 ± 6.31 (1–35)		
	< 5	69	6.70
	5–9	327	31.60
	10–14	305	29.40
	≥ 15	335	32.30
**Years working at current department (year)**	Mean ± SD = 10.28 ± 6.00 (0.3–35)		
	< 5	185	17.90
	5–9	330	31.90
	10–14	270	26.10
	≥ 15	251	24.20
**Weekly working hours (hours)**	Mean ± SD = 52.67 ± 13.89 (40–102)		
	≤ 40	392	37.80
	> 40	644	62.20
**Night shifts per month**	Mean ± SD = 6.26 ± 3.29 (0–12)		
	< 5	243	23.50
	5–9	624	60.20
	≥ 10	169	16.30
**Satisfied with work**	Dissatisfied	206	19.90
	Satisfied	830	80.10
**Experience of patient safety education**	Yes	1035	99.90
	No	1	0.10
**Experience of PCC education**	Yes	1028	99.20
	No	8	0.80
**Experience of patient safety practice**	Yes	1031	99.50
	No	5	0.50
**Experience of PCC practice**	Yes	1015	98.00
	No	21	2.00
**Patient safety incident experience**	Yes	949	91.60
	No	87	8.40
**Patient safety incident report**	Yes	684	66.00
	No	352	34.00
**Encouragement for incident reporting**	Yes	1017	98.20
	No	19	1.80
**Self-assessment of PSC**	Not good	179	17.30
	Good	857	82.7
**Self-rated patient safety in the department**	Not good	261	25.20
	Good	775	74.80

Differences in person-centered care competence, PSCp, PSMA, and PSC across general and work-related characteristics are presented in **Table 2**. Significant variations were observed across multiple demographic and professional factors (all p < 0.05). Higher PCC competence was reported by female nurses, head nurses, those working ≤40 hours per week, nurses satisfied with their work, those with PCC education and practice experience, and those reporting favorable safety conditions. PSCp varied significantly by age, education, department, position, clinical experience, night shift frequency, job satisfaction, safety-related experiences, and self-rated safety indicators, with generally higher scores among older, more experienced, and more highly educated nurses in leadership roles.

**Table 2:** Differences in person-centered care, patient safety competence, patient safety nursing activities, and patient safety culture according to general and work-related characteristics (N = 1036).

Variables	Categories	PCAT		HPEPSS		PSMA		HSOPSC	
Mean ± SD	p *Scheffe’s test*	Mean ± SD	p *Scheffe’s* *test*	Mean ± SD	p *Scheffe’s* *test*	Mean ± SD	p *Scheffe’s* *test*
**Age**	≤ 30	3.78 ± 0.36	0.617	4.17 ± 0.36	**0.004**	4.44 ± 0.35	0.839	3.93 ± 0.38	0.767
	> 30	3.77 ± 0.36		4.24 ± 0.36		4.44 ± 0.35		3.94 ± 0.34	
**Gender**	Male	3.66 ± 0.33	**0.001**	4.25 ± 0.36	0.690	4.39 ± 0.36	0.161	3.82 ± 0.38	**0.001**
	Female	3.78 ± 0.37		4.23 ± 0.36		4.44 ± 0.35		3.95 ± 0.35	
**Ethnicity**	Kinh	3.77 ± 0.36	0.940	4.23 ± 0.37	0.061	4.44 ± 0.35	**0.005**	3.93 ± 0.35	0.862
	Other	3.77 ± 0.29		4.14 ± 0.26		4.25 ± 0.37		3.92 ± 0.21	
**Religion**	No	3.78 ± 0.37	0.130	4.23 ± 0.36	0.992	4.44 ± 0.35	0.068	3.94 ± 0.35	0.111
	Yes	3.71 ± 0.32		4.23 ± 0.37		4.36 ± 0.37		3.87 ± 0.35	
**Education level**	Diploma/Associate degree	3.77 ± 0.37	0.940	4.20 ± 0.37	**0.003**	4.43 ± 0.35	0.285	3.92 ± 0.35	0.220
	Bachelor’s degree or higher	3.77 ± 0.36		4.27 ± 0.36		4.45 ± 0.36		3.95 ± 0.35	
**Marital status**	Single	3.75 ± 0.37	0.618	4.17 ± 0.39	**0.047**	4.44 ± 0.35	0.587	3.87 ± 0.41	**0.046**
	Married	3.77 ± 0.36		4.24 ± 0.36		4.43 ± 0.36		3.94 ± 0.34	
	Other	3.82 ± 0.37		4.28 ± 0.36		4.50 ± 0.29		3.98 ± 0.45	
**Working department**	Internal medicine^a^	3.77 ± 0.35	**<0.001** a,b < e	4.30 ± 0.35	**<0.001** b,c < a b < e	4.47 ± 0.37	**<0.001** b < a,d	4.02 ± 0.35	**<0.001** b,d < a b < c b < e
	Surgery^b^	3.74 ± 0.35		4.16 ± 0.35		4.34 ± 0.34		3.82 ± 0.32	
	Obstetrics–Pediatrics–Oncology^c^	3.80 ± 0.38		4.16 ± 0.40		4.41 ± 0.37		3.94 ± 0.32	
	Anesthesiology–ICU–Emergency^d^	3.71 ± 0.37		4.23 ± 0.37		4.50 ± 0.32		3.86 ± 0.39	
	Other^e^	3.91 ± 0.36		4.29 ± 0.33		4.45 ± 0.35		4.06 ± 0.28	
**Position**	Staff	3.76 ± 0.36	**0.002**	4.22 ± 0.37	**0.001**	4.44 ± 0.35	0.854	3.93 ± 0.35	0.278
	Head nurse	3.90 ± 0.33		4.36 ± 0.33		4.44 ± 0.35		3.98 ± 0.32	
**Years of clinical experience (year)**	<5^a^	3.79 ± 0.33	0.759	4.13 ± 0.32	**<0.001** a,b < d	4.43 ± 0.32	0.538	3.94 ± 0.38	0.208
	5– < 10^b^	3.77 ± 0.38		4.20 ± 0.37		4.43 ± 0.36		3.91 ± 0.37	
	10– < 15^c^	3.75 ± 0.35		4.22 ± 0.37		4.42 ± 0.36		3.92 ± 0.34	
	≥ 15^d^	3.78 ± 0.37		4.30 ± 0.35		4.46 ± 0.34		3.97 ± 0.34	
**Years working at current department (year)**	<5^a^	3.86 ± 0.34	**0.003** c,d < a	4.21 ± 0.34	0.063	4.40 ± 0.35	0.273	3.97 ± 0.33	0.197
	5– < 10^b^	3.77 ± 0.38		4.21 ± 0.37		4.43 ± 0.36		3.91 ± 0.37	
	10– < 15^c^	3.74 ± 0.34		4.23 ± 0.38		4.47 ± 0.35		3.91 ± 0.34	
	≥ 15^d^	3.75 ± 0.37		4.28 ± 0.35		4.44 ± 0.34		3.95 ± 0.35	
**Weekly working hours (hours)**	≤ 40	3.80 ± 0.36	**0.024**	4.24 ± 0.36	0.418	4.41 ± 0.36	0.084	3.98 ± 0.33	**0.002**
	> 40	3.75 ± 0.36		4.22 ± 0.37		4.45 ± 0.35		3.91 ± 0.36	
**Night shifts per month**	<5^a^	3.81 ± 0.36	0.074	4.28 ± 0.34	**0.012** c < a	4.41 ± 0.37	0.240	3.99 ± 0.34	**<0.001** c < a,b
	5– < 10^b^	3.77 ± 0.36		4.23 ± 0.37		4.45 ± 0.35		3.95 ± 0.35	
	≥ 10^c^	3.73 ± 0.38		4.17 ± 0.35		4.41 ± 0.33		3.77 ± 0.33	
**Satisfied with work**	Dissatisfied	3.62 ± 0.35	**<0.001**	4.10 ± 0.38	**<0.001**	4.39 ± 0.38	**0.045**	3.78 ± 0.39	**<0.001**
	Satisfied	3.81 ± 0.36		4.27 ± 0.35		4.45 ± 0.35		3.97 ± 0.33	
**Experience ofPCCeducation**	Yes	3.77 ± 0.36	**0.028**	4.23 ± 0.36	**0.023**	4.44 ± 0.35	0.765	3.39 ± 0.35	0.146
	No	3.49 ± 0.30		3.94 ± 0.19		4.47 ± 0.28		3.75 ± 0.31	
**Experience ofpatient safety practice**	Yes	3.77 ± 0.36	0.580	4.23 ± 0.36	0.159	4.44 ± 0.35	**0.034**	3.93 ± 0.35	0.951
	No	3.86 ± 0.30		4.46 ± 0.28		4.58 ± 0.11		3.94 ± 0.20	
**Experience ofPCC practice**	Yes	3.78 ± 0.36	**0.023**	4.24 ± 0.36	**<0.001**	4.44 ± 0.35	0.715	3.94 ± 0.35	**0.021**
	No	3.59 ± 0.29		4.01 ± 0.24		4.41 ± 0.35		3.76 ± 0.26	
**Patient safety incident experience**	Yes	3.77 ± 0.37	0.512	4.24 ± 0.37	**<0.001**	4.44 ± 0.35	0.871	3.94 ± 0.36	**<0.001**
	No	3.75 ± 0.33		4.09 ± 0.32		4.44 ± 0.34		3.81 ± 0.27	
**Patient safety incident report**	Yes	3.78 ± 0.35	0.280	4.26 ± 0.35	**0.003**	4.45 ± 0.33	0.303	3.96 ± 0.34	**<0.001**
	No	3.75 ± 0.39		4.19 ± 0.38		4.42 ± 0.39		3.87 ± 0.37	
**Encouragement for incident reporting**	Yes	3.77 ± 0.37	0.177	4.23 ± 0.36	0.501	4.44 ± 0.35	0.298	3.94 ± 0.35	0.083
	No	3.67 ± 0.26		4.18 ± 0.40		4.35 ± 0.32		3.79 ± 0.38	
**Self-assessment of PSC**	Not good	3.63 ± 0.32	**<0.001**	4.08 ± 0.35	**<0.001**	4.29 ± 0.37	**<0.001**	3.75 ± 0.32	**<0.001**
	Good	3.80 ± 0.37		4.26 ± 0.36		4.47 ± 0.34		3.97 ± 0.35	
**Self-rated patient safety in the department**	Not good	3.70 ± 0.35	**<0.001**	4.10 ± 0.31	**<0.001**	4.37 ± 0.35	**<0.001**	3.76 ± 0.30	**<0.001**
	Good	3.80 ± 0.37		4.28 ± 0.37		4.46 ± 0.35		3.99 ± 0.35	

PSMA and PSC also differed significantly across several work-related factors, particularly department, night shifts, job satisfaction, and safety perceptions, with consistently higher scores among nurses reporting more favorable work environments and safety climates.

Descriptive statistics and correlations among PCC competence PSC, PSMA, and PSC are presented in Table 3. The mean scores were 3.77 ± 0.36 for P-CAT, 4.23 ± 0.36 for H-PEPSS, 4.44 ± 0.35 for PSMA, and 3.93 ± 0.35 for HSOPSC. Pearson correlation analysis indicated that P-CAT was positively correlated with H-PEPSS (r = 0.32, p < 0.001), PSMA (r = 0.24, p < 0.001), and HSOPSC (r = 0.49, p < 0.001). H-PEPSS was also positively associated with PSMA (r = 0.35, p < 0.001) and HSOPSC (r = 0.33, p < 0.001). PSMA showed a significant positive correlation with HSOPSC (r = 0.27, p < 0.001). All correlation coefficients were in the low-to-moderate range.

**Table 3:** Descriptive analysis and correlation of person-centered care, patient safety competence, patient safety nursing activities, and patient safety culture (N = 1036).

Variable	Mean ± SD	Min – Max	PCAT	HPEPSS	PSMA	HSOPSC
r (p-value)	r (p-value)	r (p-value)	r (p-value)
**PCAT**	3.77 ± 0.36	2.77 - 4.85	1			
**HPEPSS**	4.23 ± 0.36	3.09 - 5.00	0.32 (< 0.001)	1		
**PSMA**	4.44 ± 0.35	3.25 - 5.00	0.24 (< 0.001)	0.35 (< 0.001)	1	
**HSOPSC**	3.93 ± 0.35	2.81 - 4.79	0.49 (< 0.001)	0.33 (< 0.001)	0.27 (< 0.001)	1

***Note:*** *Pearson’s correlation analysis*

Multivariable regression analyses examining predictors of PSMA and PSC (HSOPSC) are presented in Table 4. In Model 1, both PCC competence (P-CAT; β = 0.149, p < 0.001) and PSC (H-PEPSS; β = 0.274, p < 0.001) were independently associated with PSMA after adjustment for covariates, explaining 18.2% of the variance (adjusted R² = 0.173). In Model 2, P-CAT (β = 0.390, p < 0.001) and H-PEPSS (β = 0.108, p < 0.001) were significant predictors of HSOPSC, with the model accounting for 37.7% of the variance (adjusted R² = 0.366). Several work-related factors, including working department, frequent night shifts (≥10 per month), patient safety incident experience, self-assessment of PSC, and self-rated patient safety in the department, were also significantly associated with HSOPSC. In Model 3, when PSMA was included, PSMA was independently associated with HSOPSC (β = 0.102, p < 0.001), while the regression coefficients for both P-CAT (β = 0.375, p < 0.001) and H-PEPSS (β = 0.079, p = 0.006) were attenuated but remained statistically significant, indicating partial mediation. The final model explained 38.6% of the variance in PSC (adjusted R² = 0.374).

**Table 4:** Multivariable regression models predicting patient safety competence, patient safety nursing activities, and patient safety culture (N = 1036).

Variables	Model 1: PSMA β (p)	Model 2: HSOPSC (without PSMA) β (p)	Model 3: HSOPSC (with PSMA) β (p)
**PCAT**	0.149 **(<0.001)**	0.390 **(<0.001)**	0.375 **(<0.001)**
**HPEPSS**	0.274 **(<0.001)**	0.108 **(<0.001)**	0.079 **(0.006)**
**PSMA**	—	—	0.102 **(<0.001)**
**Gender** *(Female vs Male)*	—	0.033 (0.193)	0.029 (0.257)
**Marital status** *(Single = reference)*			
Married	—	0.025 (0.365)	0.028 (0.303)
Other	—	0.028 (0.299)	0.028 (0.301)
**Ethnicity** *(Kinh vs Other)*	0.081 **(0.005)**	—	—
**Working department** *(Internal = reference)*			
Surgery	−0.112 **(0.001)**	−0.157 **(<0.001)**	−0.146 **(<0.001)**
Obstetrics – Pediatrics – Oncology	−0.033 (0.308)	−0.050 (0.081)	−0.047 (0.095)
Emergency – Anesthesia & ICU	0.063 (0.057)	−0.068 **(0.026)**	−0.076 **(0.013)**
Others	−0.045 (0.147)	−0.024 (0.383)	−0.021 (0.437)
**Weekly working hours** *(>40 vs ≤ 40)*	—	−0.003 (0.905)	−0.007 (0.790)
**Night shifts per month** (< 5 *= reference)*			
5– < 10	—	0.004 (0.898)	0.000 (0.993)
≥ 10	—	−0.139 **(<0.001)**	−0.139 **(<0.001)**
**Experience ofpatient safety practice** (*Yes vs No)*	−0.011 (0.705)	—	—
**Experience ofPCC practice** (*Yes vs No)*	—	0.012 (0.642)	0.014 (0.568)
**Patient safety incident experience** (*Yes vs No)*	—	0.056 **(0.034)**	0.061 **(0.021)**
**Patient safety incident report** (*Yes vs No)*	—	0.039 (0.149)	0.036 (0.175)
**Satisfied with work** *(Satisfied vs Dissatisfied)*	−0.046 (0.129)	0.038 (0.158)	0.042 (0.108)
**Self-assessment of PSC** *(Good vs Not good)*	0.119 **(<0.001)**	0.060 **(0.029)**	0.048 (0.081)
**Self-rated patient safety in the department** *(Good vs Not good)*	0.005 (0.875)	0.181 **(<0.001)**	0.179 **(<0.001)**
	R^2^ = 0.182, Adjusted R^2^ = 0.173, F = 20.73, **p < 0.001**	R^2^ = 0.377, Adjusted R^2^ = 0.366, F = 34.24, **p < 0.001**	R^2^ = 0.386, Adjusted R^2^ = 0.374, F = 33.59, **p < 0.001**

Note: β: standardized regression coefficient; PSC: patient safety culture; PSMA: patient safety management activities. Covariates included sociodemographic and professional variables as described in the Methods section.Full regression results, including unstandardized coefficients, standard errors, and variance inflation factors (VIF), are provided in Supplementary S1 Table.

Mediation analysis using bootstrapping with 5,000 resamples demonstrated that PSMA partially mediated the relationships between both PCC competence and PSC, as well as PSCp and PSC (Table 5). For the pathway from PCC competence (P-CAT) to PSC (HSOPSC), the total effect was statistically significant (β = 0.4055, 95% CI: 0.3561–0.4549). After accounting for PSMA, the direct effect remained significant (β = 0.3790, 95% CI: 0.3288–0.4291), while the indirect effect through PSMA was also significant (β = 0.0265, 95% CI: 0.0143–0.0404), indicating partial mediation. Similarly, PSCp (H-PEPSS) showed a significant total effect on PSC (β = 0.2125, 95% CI: 0.1577–0.2673). When PSMA was included in the model, the direct effect was attenuated but remained significant (β = 0.1628, 95% CI: 0.1057–0.2199), and a significant indirect effect via PSMA was observed (β = 0.0497, 95% CI: 0.0304–0.0711), also supporting partial mediation. In both models, the indirect effects were statistically significant as the bias-corrected confidence intervals did not include zero, suggesting that PSMA represent an important behavioral pathway through which nurses’ competencies are associated with PSC. The proportion of the total effect mediated by PSMA was approximately 6.5% for person-centered care competence and 23.4% for patient safety competence.

**Table 5:** Mediation effects of patient safety management activities in the relationships between professional competencies and patient safety culture (Bootstrapping with 5,000 resamples) (N = 1036).

Pathway	Effect type	β	95% CI	p-value	% mediated
**P-CAT → PSMA → HSOPSC**	Total effect (c)	0.4055	0.3561–0.4549	<0.001	—
	Direct effect (c′)	0.3790	0.3288–0.4291	<0.001	—
	Indirect effect (a × b)	0.0265	0.0143–0.0404	—	6.5
**H-PEPSS → PSMA → HSOPSC**	Total effect (c)	0.2125	0.1577–0.2673	<0.001	—
	Direct effect (c′)	0.1628	0.1057–0.2199	<0.001	—
	Indirect effect (a × b)	0.0497	0.0304–0.0711	—	23.4

***Note:*** *Indirect effects were estimated using bias-corrected bootstrapping with 5,000 resamples. Indirect effects were considered statistically significant when the 95% confidence interval did not include zero. p-values are not reported for indirect effects, in accordance with recommendations for bootstrap mediation analysis.*

## Discussion

### Levels of PCC competence, patient safety competence, patient safety management activities, and patient safety culture

The present findings suggest that nurses reported moderate-to-high levels of PCC competence, PSCp, and PSMA, whereas PSC was perceived at a moderate level. This pattern—relatively strong individual competence alongside only moderate organizational safety culture—has been observed in several hospital settings, particularly in low- and middle-income countries [28–30]. This similarity may be explained by the influence of systemic and organizational constraints that limit the translation of individual competencies into consistent safety culture across healthcare settings.

This divergence may reflect the distinction between individual capability and collective organizational climate. While professional competence is largely shaped by education, training, and clinical exposure, safety culture is embedded within institutional structures, leadership behaviors, communication norms, and reporting systems. Evidence from validation studies of PSCp instruments suggests that nurses often rate interpersonal domains, such as teamwork and communication, more positively than system-level dimensions related to organizational safety infrastructure [9,31].

The relatively high level of engagement in PSMA observed in this study further supports the interpretation that nurses consistently perform protocol-driven safety behaviors embedded in daily workflows. Similar findings have been reported in studies using validated safety activity scales [22], In Vietnamese hospital settings, previous studies have also reported generally positive engagement with patient safety practices and safety culture among nurses [32].

However, despite these favorable behavioral indicators, PSC remained moderate. This finding may differ from studies conducted in more resource-rich healthcare systems, where stronger institutional support and safety infrastructures may facilitate a closer alignment between individual competencies and organizational safety culture. This gap may reflect structural and organizational constraints, including limited resources, staffing challenges, and less developed safety systems, which may hinder the consistent translation of individual competencies into organizational safety culture. [2]. Vietnamese hospital studies have similarly highlighted variability across culture domains, particularly in staffing adequacy, non-punitive response to error, and open communication [32].

From a conceptual perspective, these findings align with a structure–process–outcome model, in which professional competence represents a structural input, patient safety management activities reflect care processes, and PSC constitutes an organizational outcome [14]. Global patient safety policy frameworks emphasize that improvements in competence and safety practices require supportive leadership, learning systems, and reporting environments to generate sustained cultural change [4,5].

These findings underscore a structural tension between individual professional preparedness and organizational readiness for patient safety within tertiary hospital settings.

### Direct and indirect relationships among competencies, safety activities, and patient safety culture

Beyond descriptive findings, this study examined how PCC competence and PSCp are linked to PSC through both direct and indirect pathways. Both competence domains were significantly associated with PSC, and PSMA partially mediated these relationships. The significant indirect effects observed in the bootstrapped mediation analyses suggest that frontline safety behaviors may represent a potential pathway through which professional competencies are associated with broader organizational safety culture.

Although the standardized association between patient safety management activities and patient safety culture was modest (β = 0.102), this finding remains practically meaningful. In complex healthcare systems, even small improvements in frontline safety behaviors may contribute to observable differences in perceived safety culture over time. Given that patient safety management activities reflect routine and modifiable practices embedded in daily clinical workflows, strengthening these activities may represent a feasible strategy to support improvements in nurses’ perceptions of patient safety culture.

In this study, patient safety culture was assessed at the individual level, reflecting nurses’ perceptions rather than aggregated organizational-level safety climate. However, consistent with the cross-sectional design of this study, these findings should be interpreted as associations rather than causal effects.

The direct association between PSCp and PSC observed in this study is consistent with accumulating international evidence demonstrating that higher levels of clinical competence are associated with stronger safety culture across hospital settings [11]. This consistency may reflect the universal role of core competencies, such as teamwork, communication, and risk management, in shaping shared safety perceptions across diverse healthcare systems. Multicenter cross-sectional research has further shown that PSCp is positively associated with safety culture and inversely associated with adverse events, suggesting that competence may contribute not only to perceptions but also to measurable safety outcomes [33]. These findings reinforce the interpretation that competence in teamwork, communication, and risk management contributes to shaping shared safety norms within clinical environments.

Importantly, the mediating role of PSMA supports a competence–behavior–culture pathway. PSMA represent observable and routine safety practices embedded in daily nursing workflows, including patient identification, medication safety, infection prevention, and fall prevention. Evidence indicates that safety competence and safety culture jointly predict safety nursing activities [34], and that safety culture may mediate relationships between professional attributes and safety-related outcomes [35]. The present findings extend this body of work by suggesting that frontline safety behaviors function as a practical conduit through which competence is operationalized and subsequently reflected in collective safety perceptions.

The partial nature of the mediation effect is theoretically meaningful. While engagement in safety management activities explains part of the association between competence and PSC, a substantial direct effect remained. This suggests that competencies may influence safety culture not only through behavioral enactment but also through cognitive and attitudinal mechanisms, such as risk awareness, professional responsibility, and shared commitment to safety. At the same time, extensive evidence indicates that safety culture is shaped by broader organizational determinants, including leadership engagement, communication climate, staffing adequacy, and non-punitive responses to error [30,36].

Within the Vietnamese context, national studies have similarly demonstrated that managerial support, communication openness, and reporting systems significantly influence safety culture perceptions [28,32]. These contextual findings support the interpretation that individual competence, although necessary, is insufficient in isolation without supportive organizational conditions that enable consistent and psychologically safe enactment of safety practices.

Taken together, the present findings support an integrated and multidimensional pathway linking nurses’ competencies, frontline safety behaviors, and patient safety culture. Strengthening PCC competence and PSCp may be associated with improvements in safety culture both directly and indirectly through sustained engagement in PSMA. However, durable cultural transformation likely requires simultaneous reinforcement of leadership practices, organizational learning systems, and supportive work environments to align individual capability with institutional readiness for patient safety.

This underscores the importance of aligning individual capability with organizational readiness to achieve sustainable improvements in patient safety culture. These findings may inform the design of interventions that integrate competency development with system-level support for patient safety practices. Such interventions may include competency-based training programs, structured safety protocols, and organizational policies that promote a supportive and non-punitive safety environment. Given the cross-sectional design, these findings should be interpreted as associations rather than causal relationships.

### Limitations

This study has several limitations. First, the cross-sectional design precludes causal inference; therefore, the mediation results should be interpreted as indicating indirect associations rather than causal pathways. Second, all variables were measured using self-reported questionnaires, which may introduce the risk of common method bias. Third, the use of convenience sampling at the hospital selection stage may limit the generalizability of the findings. Fourth, one instrument demonstrated relatively low internal consistency, although it remained within an acceptable range for exploratory research. Fifth, although internal consistency reliability was assessed, further psychometric validation, including confirmatory factor analysis, was not performed and should be considered in future studies. Sixth, measurement invariance across subgroups (e.g., gender, department, or hospital) was not examined, which may limit the comparability of the constructs across different participant groups. Seventh, HSOPSC was analyzed at the individual level, reflecting nurses’ perceptions of patient safety culture, and was not aggregated to the unit or hospital level, which may limit interpretation of organizational-level safety climate.

## Conclusions

This study found that person-centered care competence and patient safety competence were associated with patient safety culture among nurses in tertiary hospitals. Patient safety management activities partially mediated these relationships, suggesting that frontline safety behaviors may represent a pathway through which competencies are translated into organizational safety culture. These findings highlight the potential role of patient safety management activities as a mechanism linking nursing competencies to patient safety culture. Strengthening both competencies and organizational systems that support safety practices may be important for improving patient safety culture. However, causal relationships cannot be established due to the cross-sectional design. Further longitudinal or experimental studies are needed to confirm these relationships and evaluate system-level interventions.

## Supporting information

S1 Dataset Mediating effects of patient safety management activities among nurses dataset. (XLS)

S1 File Coefficients of Regession results. (DOC)

S2 File Cronbach alpha and McDonalds omega. (PDF)

## References

[1] Weaver SJ, Lubomksi LH, Wilson RF, Pfoh ER, Martinez KA, Dy SM. Promoting a culture of safety as a patient safety strategy: a systematic review. Ann Intern Med. 2013;158(5 Pt 2):369–74. doi: 10.7326/0003-4819-158-5-201303051-00002 23460092 PMC4710092

[2] Halligan M, Zecevic A. Safety culture in healthcare: a review of concepts, dimensions, measures and progress. BMJ Qual Saf. 2011;20(4):338–43. doi: 10.1136/bmjqs.2010.040964 21303770

[3] Okuyama A, Wagner C, Bijnen B. Speaking up for patient safety by hospital-based health care professionals: a literature review. BMC Health Serv Res. 2014;14:61–8. doi: 10.1186/1472-6963-14-61 24507747 PMC4016383

[4] WHO. Global patient safety action plan 2021-2030. 2021. https://www.who.int/teams/integrated-health-services/patient-safety/policy/global-patient-safety-action-plan

[5] WHO. Global patient safety action plan 2021–2030: implementation report 2024. 2024.

[6] WHO. WHO global strategy on people-centred and integrated health services. 2015. 10.1111/ajr.12209 26009975

[7] Santana MJ, Manalili K, Jolley RJ, Zelinsky S, Quan H, Lu M. How to practice person-centred care: A conceptual framework. Health Expect. 2018;21(2):429–40. doi: 10.1111/hex.12640 29151269 PMC5867327

[8] WHO. People-centred care in health systems: improving quality and safety. 2022.

[9] Ginsburg L, Castel E, Tregunno D, Norton PG. The H-PEPSS: an instrument to measure health professionals’ perceptions of patient safety competence at entry into practice. BMJ Qual Saf. 2012;21(8):676–84. doi: 10.1136/bmjqs-2011-000601 22562876 PMC3402748

[10] Alquwez N. Examining the Influence of Workplace Incivility on Nurses’ Patient Safety Competence. J Nurs Scholarsh. 2020;52(3):292–300. doi: 10.1111/jnu.12553 32267623

[11] Zaitoun RA, Said NB, de Tantillo L. Clinical nurse competence and its effect on patient safety culture: a systematic review. BMC Nurs. 2023;22(1):173. doi: 10.1186/s12912-023-01305-w 37208727 PMC10196295

[12] Ajzen I. The Theory of Planned Behavior. Organ Behav Hum Decis Process. 1991;50(2):179–211. doi: 10.1016/0749-5978(91)90020-T

[13] Neal A, Griffin MA. A study of the lagged relationships among safety climate, safety motivation, safety behavior, and accidents at the individual and group levels. J Appl Psychol. 2006;91(4):946–53. doi: 10.1037/0021-9010.91.4.946 16834517

[14] Donabedian A. Evaluating the quality of medical care. Milbank Q. 2005;83(4):691–729. doi: 10.1111/j.1468-0009.2005.00397.x 16279964 PMC2690293

[15] Reason J. Human error: models and management. BMJ. 2000;320(7237):768–70. doi: 10.1136/bmj.320.7237.768 10720363 PMC1117770

[16] von Elm E, Altman DG, Egger M, Pocock SJ, Gøtzsche PC, Vandenbroucke JP, et al. The Strengthening the Reporting of Observational Studies in Epidemiology (STROBE) statement: guidelines for reporting observational studies. J Clin Epidemiol. 2008;61(4):344–9. doi: 10.1016/j.jclinepi.2007.11.008 18313558

[17] Cohen J. Statistical power analysis for the behavioral sciences. 2nd ed ed. Hillsdale, NJ: Lawrence Erlbaum Associates. 1988.

[18] Cochran WG. Sampling techniques. 3rd ed. New York: John Wiley & Sons. 1977.

[19] WHO. Process of translation and adaptation of instruments. 2018.

[20] Beaton DE, Bombardier C, Guillemin F, Ferraz MB. Guidelines for the process of cross-cultural adaptation of self-report measures. Spine (Phila Pa 1976). 2000;25(24):3186–91. doi: 10.1097/00007632-200012150-00014 11124735

[21] Edvardsson D, Fetherstonhaugh D, Nay R, Gibson S. Development and initial testing of the Person-centered Care Assessment Tool (P-CAT). Int Psychogeriatr. 2010;22(1):101–8. doi: 10.1017/S1041610209990688 19631005

[22] Park H-H, Kim S. A Structural Equation Model of Nurses’ Patient Safety Management Activities. J Korean Acad Nurs Adm. 2019;25(2):63. doi: 10.11111/jkana.2019.25.2.63

[23] World Health Organization. Patient safety curriculum guide: multi-professional edition. 2011.

[24] Hall KK, Shoemaker-Hunt S, Hoffman L, Richard S, Gall E, Schoyer E. Making Healthcare Safer III: A Critical Analysis of Existing and Emerging Patient Safety Practices. Rockville (MD): Agency for Healthcare Research and Quality (US). 2020. 32255576

[25] Sorra JS, DN DN. Hospital survey on patient safety culture: User’s guide. 2010. https://www.ahrq.gov/sops/surveys/hospital/index.html 10.1186/1472-6963-10-199 PMC2912897 20615247

[26] Cohen J, West SG, Aiken LS. Applied Multiple Regression/Correlation Analysis for the Behavioral Sciences. 3rd ed ed. Mahwah, NJ: Lawrence Erlbaum Associates. 2003.

[27] Preacher KJ, Hayes AF. Asymptotic and resampling strategies for assessing and comparing indirect effects in multiple mediator models. Behav Res Methods. 2008;40(3):879–91. doi: 10.3758/brm.40.3.879 18697684

[28] Thu NTH, Anh BTM, Ha NTT, Tien DNT, Giang PH, Nga TT, et al. Health staff perceptions of patient safety and associated factors in hospitals in Vietnam. Front Public Health. 2023;11:1149667. doi: 10.3389/fpubh.2023.1149667 37965513 PMC10641002

[29] Tran TNH, Pham QT, Tran LH, Vu TA, Nguyen MT, Pham HT, et al. Comparison of Perceptions About Patient Safety Culture Between Physicians and Nurses in Public Hospitals in Vietnam. Risk Manag Healthc Policy. 2022;15:1695–704. doi: 10.2147/RMHP.S373249 36097561 PMC9464021

[30] Atinafu D, Getaneh G, Setotaw G. Assessment of patient safety culture and associated factors among healthcare professionals in public hospitals of Bahir Dar City, Northwest Ethiopia: A mixed-methods study. PLoS One. 2024;19(11):e0313321. doi: 10.1371/journal.pone.0313321 39509370 PMC11542836

[31] Boloré S, Sovet L, Guirimand N. Health professionals’ perceptions of patient safety competencies: psychometric properties of the French version of the H-PEPSS in France and Switzerland. BMC Med Educ. 2023;23(1):905. doi: 10.1186/s12909-023-04893-y 38031021 PMC10688088

[32] Ha TTN, Thanh PQ, Huong TL, Anh VT, Tu NM, Tien PH, et al. Nurses’ perceptions about patient safety culture in public hospital in Vietnam. Appl Nurs Res. 2023;69:151650. doi: 10.1016/j.apnr.2022.151650 36635007

[33] Hafezi A, Babaii A, Aghaie B, Abbasinia M. The relationship between patient safety culture and patient safety competency with adverse events: a multicenter cross-sectional study. BMC Nurs. 2022;21(1):292. doi: 10.1186/s12912-022-01076-w 36319970 PMC9628064

[34] Kim JJ, Mi JH. Effect of patient safety culture and patient safety competence on safety nursing activity among nurses working in anesthetic and recovery rooms. J Korean Clin Nurs Res. 2020;26(2):164–74. 10.22650/JKCNR.2020.26.2.164

[35] Park MN, Roh YS. Emergency department nurses’ reflective thinking and patient safety competency: The mediating effect of patient safety culture. Appl Nurs Res. 2024;80:151856. doi: 10.1016/j.apnr.2024.151856 39617606

[36] Tawfik DS, Adair KC, Palassof S, Sexton JB, Levoy E, Frankel A. Joint Commission Journal on Quality and Patient Safety. 2023;49(3):156–65. doi: 10.1016/j.jcjq.2022.12.006 36658090 PMC9974844

