# Supplementary material for: Patient safety management activities partially mediate nursing competences and patient safety culture in Vietnam
Source: PLoS One. 2026 Jul 13;21(7):e0345533. doi: 10.1371/journal.pone.0345533 (PMC13362141; doi:10.1371/journal.pone.0345533)
Supplement: S1 File — (DOC) [file pone.0345533.s002.doc]

**Model 1: PSMA**

| **Coefficients^a^** | | | | | | | | | | | |
| --- | --- | --- | --- | --- | --- | --- | --- | --- | --- | --- | --- |
| Model | | Unstandardized Coefficients | | Standardized Coefficients | t | Sig. | Correlations | | | Collinearity Statistics | |
|  |  | B | Std. Error | Beta |  |  | Zero-order | Partial | Part | Tolerance | VIF |
| 1 | (Constant) | 2,610 | ,213 |  | 12,241 | ,000 |  |  |  |  |  |
|  | PCAT | ,144 | ,030 | ,149 | 4,851 | ,000 | ,240 | ,150 | ,137 | ,850 | 1,176 |
|  | HPEPSS | ,265 | ,030 | ,274 | 8,794 | ,000 | ,352 | ,265 | ,249 | ,825 | 1,211 |
|  | Kinh | ,176 | ,062 | ,081 | 2,821 | ,005 | ,087 | ,088 | ,080 | ,978 | 1,023 |
|  | Surgery | -,096 | ,028 | -,112 | -3,367 | ,001 | -,146 | -,105 | -,095 | ,724 | 1,380 |
|  | Obstetrics – Pediatrics – Oncology | -,033 | ,032 | -,033 | -1,019 | ,308 | -,036 | -,032 | -,029 | ,775 | 1,290 |
|  | ICU | ,053 | ,028 | ,063 | 1,902 | ,057 | ,090 | ,059 | ,054 | ,734 | 1,362 |
|  | Others | -,052 | ,036 | -,045 | -1,453 | ,147 | ,009 | -,045 | -,041 | ,815 | 1,227 |
|  | Experience of patient safety practice (Yes) | -,055 | ,145 | -,011 | -,378 | ,705 | -,029 | -,012 | -,011 | ,986 | 1,014 |
|  | Satisfied with work | -,041 | ,027 | -,046 | -1,521 | ,129 | ,062 | -,047 | -,043 | ,880 | 1,137 |
|  | Self-assessment of PSC (Good) | ,111 | ,029 | ,119 | 3,842 | ,000 | ,187 | ,119 | ,109 | ,833 | 1,200 |
|  | Self-rated patient safety in the department (Good) | ,004 | ,025 | ,005 | ,157 | ,875 | ,115 | ,005 | ,004 | ,838 | 1,193 |
| a. Dependent Variable: PSMA | | | | | | | | | | | |

**Model 2: HSOPSS without PSMA**

| **Coefficients^a^** | | | | | | | | | | | |
| --- | --- | --- | --- | --- | --- | --- | --- | --- | --- | --- | --- |
| Model | | Unstandardized Coefficients | | Standardized Coefficients | t | Sig. | Correlations | | | Collinearity Statistics | |
|  |  | B | Std. Error | Beta |  |  | Zero-order | Partial | Part | Tolerance | VIF |
| 2 | (Constant) | 1,795 | ,140 |  | 12,805 | ,000 |  |  |  |  |  |
|  | PCAT | ,377 | ,026 | ,390 | 14,450 | ,000 | ,486 | ,413 | ,358 | ,842 | 1,188 |
|  | HPEPSS | ,104 | ,027 | ,108 | 3,922 | ,000 | ,333 | ,122 | ,097 | ,809 | 1,236 |
|  | Female | ,039 | ,030 | ,033 | 1,304 | ,193 | ,107 | ,041 | ,032 | ,936 | 1,068 |
|  | Married | ,023 | ,025 | ,025 | ,906 | ,365 | ,060 | ,028 | ,022 | ,822 | 1,216 |
|  | Other | ,061 | ,059 | ,028 | 1,038 | ,299 | ,024 | ,033 | ,026 | ,850 | 1,176 |
|  | Surgery | -,134 | ,025 | -,157 | -5,369 | ,000 | -,168 | -,166 | -,133 | ,720 | 1,389 |
|  | Obstetrics – Pediatrics – Oncology | -,050 | ,029 | -,050 | -1,747 | ,081 | ,004 | -,055 | -,043 | ,751 | 1,332 |
|  | ICU | -,058 | ,026 | -,068 | -2,224 | ,026 | -,111 | -,070 | -,055 | ,651 | 1,536 |
|  | Others | -,028 | ,032 | -,024 | -,873 | ,383 | ,120 | -,027 | -,022 | ,807 | 1,239 |
|  | Night shifts per month (5–<10) | ,003 | ,023 | ,004 | ,128 | ,898 | ,069 | ,004 | ,003 | ,589 | 1,697 |
|  | Night shifts per month (≥ 10) | -,132 | ,031 | -,139 | -4,207 | ,000 | -,202 | -,131 | -,104 | ,564 | 1,772 |
|  | Weekly working hours | -,002 | ,020 | -,003 | -,120 | ,905 | -,098 | -,004 | -,003 | ,794 | 1,260 |
|  | Experience of PCC practice | ,029 | ,063 | ,012 | ,464 | ,642 | ,072 | ,015 | ,011 | ,944 | 1,059 |
|  | Patient safety incident experience | ,071 | ,034 | ,056 | 2,118 | ,034 | ,106 | ,066 | ,052 | ,870 | 1,150 |
|  | Patient safety incident report | ,029 | ,020 | ,039 | 1,443 | ,149 | ,122 | ,045 | ,036 | ,853 | 1,172 |
|  | Satisfied with work | ,033 | ,023 | ,038 | 1,414 | ,158 | ,218 | ,044 | ,035 | ,870 | 1,150 |
|  | Self-assessment of PSC | ,056 | ,026 | ,060 | 2,184 | ,029 | ,244 | ,068 | ,054 | ,801 | 1,249 |
|  | Self-rated patient safety in the department | ,146 | ,022 | ,181 | 6,682 | ,000 | ,292 | ,205 | ,165 | ,839 | 1,192 |
| a. Dependent Variable: HSOPSS **(without PSMA)** | | | | | | | | | | | |

| **Coefficients^a^** | | | | | | | | | | | |
| --- | --- | --- | --- | --- | --- | --- | --- | --- | --- | --- | --- |
| Model | | Unstandardized Coefficients | | Standardized Coefficients | t | Sig. | Correlations | | | Collinearity Statistics | |
|  |  | B | Std. Error | Beta |  |  | Zero-order | Partial | Part | Tolerance | VIF |
| 3 | (Constant) | 1,519 | ,158 |  | 9,635 | ,000 |  |  |  |  |  |
|  | PCAT | ,362 | ,026 | ,375 | 13,849 | ,000 | ,486 | ,399 | ,341 | ,824 | 1,214 |
|  | HPEPSS | ,076 | ,027 | ,079 | 2,763 | ,006 | ,333 | ,086 | ,068 | ,748 | 1,338 |
|  | Female | ,033 | ,029 | ,029 | 1,135 | ,257 | ,107 | ,036 | ,028 | ,934 | 1,071 |
|  | Married | ,026 | ,025 | ,028 | 1,031 | ,303 | ,060 | ,032 | ,025 | ,822 | 1,217 |
|  | Other | ,061 | ,059 | ,028 | 1,036 | ,301 | ,024 | ,032 | ,025 | ,850 | 1,176 |
|  | Surgery | -,125 | ,025 | -,146 | -5,023 | ,000 | -,168 | -,156 | -,124 | ,713 | 1,402 |
|  | Obstetrics – Pediatrics – Oncology | -,047 | ,028 | -,047 | -1,669 | ,095 | ,004 | -,052 | -,041 | ,751 | 1,332 |
|  | ICU | -,064 | ,026 | -,076 | -2,475 | ,013 | -,111 | -,077 | -,061 | ,648 | 1,542 |
|  | Others | -,024 | ,031 | -,021 | -,778 | ,437 | ,120 | -,024 | -,019 | ,807 | 1,240 |
|  | Night shifts per month (5–<10) | ,000 | ,023 | ,000 | -,009 | ,993 | ,069 | ,000 | ,000 | ,589 | 1,699 |
|  | Night shifts per month (≥ 10) | -,132 | ,031 | -,139 | -4,241 | ,000 | -,202 | -,132 | -,104 | ,564 | 1,772 |
|  | Weekly working hours | -,005 | ,020 | -,007 | -,267 | ,790 | -,098 | -,008 | -,007 | ,793 | 1,261 |
|  | Experience of PCC practice | ,036 | ,063 | ,014 | ,571 | ,568 | ,072 | ,018 | ,014 | ,943 | 1,060 |
|  | Patient safety incident experience | ,077 | ,033 | ,061 | 2,308 | ,021 | ,106 | ,072 | ,057 | ,868 | 1,152 |
|  | Patient safety incident report | ,027 | ,020 | ,036 | 1,357 | ,175 | ,122 | ,043 | ,033 | ,853 | 1,173 |
|  | Satisfied with work | ,037 | ,023 | ,042 | 1,607 | ,108 | ,218 | ,050 | ,039 | ,867 | 1,153 |
|  | Self-assessment of PSC | ,045 | ,026 | ,048 | 1,745 | ,081 | ,244 | ,055 | ,043 | ,790 | 1,266 |
|  | Self-rated patient safety in the department | ,145 | ,022 | ,179 | 6,678 | ,000 | ,292 | ,205 | ,164 | ,839 | 1,192 |
|  | PSMA | ,101 | ,027 | ,102 | 3,736 | ,000 | ,274 | ,116 | ,092 | ,814 | 1,229 |
| a. Dependent Variable: HSOPSS | | | | | | | | | | | |
