# Supplementary material for: Patient safety management activities partially mediate nursing competences and patient safety culture in Vietnam
Source: PLoS One. 2026 Jul 13;21(7):e0345533. doi: 10.1371/journal.pone.0345533 (PMC13362141; doi:10.1371/journal.pone.0345533)
Supplement: S2 File — (PDF) [file pone.0345533.s003.pdf]

Results

Reliability Analysis

Scale Reliability Statistics

	Cronbach's α	McDonald's ω
**scale**	0.662	0.712

Note. items 'F3', 'F4', 'F5', 'F6', 'F11', and 'F13' correlate negatively with the total scale and probably should be reversed

[3]

Item Reliability Statistics

	If item dropped	
	Cronbach's α	McDonald's ω
**F1**	0.634	0.684
**F2**	0.629	0.680
**F3**	0.656	0.705
**F4**	0.671	0.710
**F5**	0.650	0.702
**F6**	0.651	0.701
**F7**	0.617	0.687
**F8**	0.597	0.679
**F9**	0.646	0.702
**F10**	0.616	0.685
**F11**	0.647	0.698
**F12**	0.647	0.705
**F13**	0.691	0.727

Reliability Analysis

Scale Reliability Statistics

	Cronbach's α	McDonald's ω
**scale**	0.898	0.910

[3]

Item Reliability Statistics

	If item dropped	
	**Cronbach's α**	**McDonald's ω**
**tD1**	0.895	0.908
**D2**	0.898	0.910
**D3**	0.893	0.905
**D4**	0.892	0.905
**D5**	0.897	0.908
**D6**	0.893	0.906
**D7**	0.893	0.905
**D8**	0.892	0.905
**D9**	0.893	0.905
**D10**	0.892	0.904
**D11**	0.892	0.904
**D12**	0.892	0.905
**D13**	0.895	0.908
**D14**	0.892	0.906
**D15**	0.893	0.906
**D16**	0.892	0.904
**D17**	0.893	0.906
**D18**	0.902	0.911
**D19**	0.892	0.905
**D20**	0.899	0.910
**D21**	0.892	0.905
**D22**	0.892	0.905
**D23**	0.893	0.906

Reliability Analysis

Scale Reliability Statistics

	**Cronbach's α**	**McDonald's ω**
**scale**	0.863	0.893

[3]

Item Reliability Statistics

	If item dropped	
	Cronbach's α	McDonald's ω
**E1**	0.862	0.892
**E2**	0.859	0.888
**E3**	0.858	0.886
**E4**	0.859	0.887
**E5**	0.859	0.890
**E6**	0.856	0.889
**E7**	0.855	0.889
**E8**	0.862	0.891
**E9**	0.857	0.890
**E10**	0.859	0.889
**E11**	0.871	0.893
**E12**	0.858	0.887
**E13**	0.858	0.887
**E14**	0.858	0.891
**E15**	0.858	0.887
**E16**	0.857	0.885
**E17**	0.858	0.886
**E18**	0.857	0.885
**E19**	0.858	0.887
**E20**	0.858	0.889
**E21**	0.858	0.890
**E22**	0.858	0.890
**E23**	0.856	0.889
**E24**	0.855	0.887

Reliability Analysis

Scale Reliability Statistics

	Cronbach's α	McDonald's ω
**scale**	0.889	0.911

[3]

Item Reliability Statistics

	If item dropped	
	Cronbach's α	McDonald's ω
**H1**	0.887	0.908
**H2**	0.887	0.909
**H3**	0.887	0.909
**H4**	0.886	0.908
**H5**	0.891	0.913
**H6**	0.887	0.908
**H7**	0.889	0.911
**H8**	0.886	0.910
**H9**	0.888	0.911
**H10**	0.887	0.910
**H11**	0.886	0.908
**H12**	0.885	0.910
**H13**	0.886	0.908
**H14**	0.884	0.909
**H15**	0.894	0.912
**H16**	0.888	0.911
**H17**	0.886	0.910
**H18**	0.885	0.907
**H19**	0.886	0.907
**H20**	0.886	0.908
**H21**	0.884	0.909
**H22**	0.884	0.908
**H23**	0.886	0.907
**H24**	0.886	0.908
**H25**	0.886	0.908
**H26**	0.892	0.912
**H27**	0.885	0.907
**H28**	0.886	0.910
**H29**	0.887	0.909
**H30**	0.888	0.910
**H31**	0.887	0.910
**H32**	0.885	0.907
**H33**	0.886	0.910
**H34**	0.886	0.910
**H35**	0.885	0.908
**H36**	0.883	0.908
**H37**	0.884	0.909

Item Reliability Statistics

**H38**	0.886	0.910
**H39**	0.885	0.907
**H40**	0.887	0.910
**H41**	0.885	0.907
**H42**	0.885	0.909

References

[1] The jamovi project (2024). *jamovi*. (Version 2.6) [Computer Software]. Retrieved from https://www.jamovi.org.

[2] R Core Team (2024). *R: A Language and environment for statistical computing*. (Version 4.4) [Computer software]. Retrieved from https://cran.r-project.org. (R packages retrieved from CRAN snapshot 2024-08-07).

[3] Revelle, W. (2023). *psych: Procedures for Psychological, Psychometric, and Personality Research*. [R package]. Retrieved from https://cran.r-project.org/package=psych.
